# Opposite effects of choice history and evidence history resolve a paradox of sequential choice bias

**DOI:** 10.1167/jov.20.12.9

**Published:** 2020-11-19

**Authors:** Ella Bosch, Matthias Fritsche, Benedikt V. Ehinger, Floris P. de Lange

**Affiliations:** 1 Donders Institute for Brain, Cognition and Behaviour, Radboud University, Nijmegen, The Netherlands

**Keywords:** choice repetition, sequential choice bias, serial dependence, confidence, decision-making

## Abstract

Perceptual decisions are biased toward previous decisions. Earlier research suggests that this choice repetition bias is increased after previous decisions of high confidence, as inferred from response time measures (Urai, Braun, & Donner, 2017), but also when previous decisions were based on weak sensory evidence (Akaishi, Umeda, Nagase, & Sakai, 2014). As weak sensory evidence is typically associated with low confidence, these previous findings appear conflicting. To resolve this conflict, we set out to investigate the effect of decision confidence on choice repetition more directly by measuring explicit confidence ratings in a motion coherence discrimination task. Moreover, we explored how choice and evidence history jointly affect subsequent perceptual choices. We found that participants were more likely to repeat previous choices of high subjective confidence, as well as previous fast choices, confirming the boost of choice repetition with decision confidence. Furthermore, we discovered that current choices were biased away from the previous evidence direction and that this effect grew with previous evidence strength. These findings point toward simultaneous biases of choice repetition, modulated by decision confidence, and evidence adaptation, modulated by the strength of evidence, which bias current perceptual decisions in opposite directions.

## Introduction

Perceptual decisions not only are based on current sensory evidence but are also influenced by the choice history. Across a wide range of perceptual decision-making tasks, observers tend to repeat their decisions more than is expected by chance (Abrahamyan, Silva, Dakin, Carandini, & Gardner, 2016; Akaishi, Umeda, Nagase, & Sakai, 2014; Braun, Urai, & Donner, 2018; Fischer & Whitney, 2014; Fritsche, Mostert, & de Lange, 2017; Fründ, Wichmann, & Macke, 2014; St. John-Saaltink, Kok, Lau, & de Lange, 2016; Urai, Braun, & Donner, 2017, Urai, de Gee, Tsetsos, & Donner, 2019). This choice repetition bias occurs not only in human perceptual decision-making but also in that of monkeys (Gold, Law, Connolly, & Bennur, 2008) and rodents (Busse et al., 2011; Odoemene, Pisupati, Nguyen, & Churchland, 2018), and it has been found in other domains of human decision-making, such as free-choice tasks and economic decisions (Allefeld, Soon, Bogler, Heinzle, & Haynes, 2013; Padoa-Schioppa, 2013). In summary, choice history biases appear to be a general feature of decision-making.

Why does choice history bias occur? This is an especially good question given that, in most laboratory experiments, stimuli are uncorrelated across trials. Repeating previous choices is consequently detrimental to task performance; choice history biases are maladaptive in the contexts of such tasks. However, they are likely adaptive in natural conditions, as our environment usually remains relatively stable over short timescales (Dong & Atick, 1995; Simoncelli & Olshausen, 2001). Crucially, observers can exploit this stability by leveraging information from the recent past in order to stabilize perceptual decisions against disturbing factors, such as noise (Cicchini, Mikellidou, & Burr, 2017; Fischer & Whitney, 2014). Temporally smoothing internal representations in this manner would manifest itself in a tendency to repeat previous decisions. Choice history biases that persist despite uncorrelated input may be a consequence of our prior expectation that our environment tends to be temporally correlated.

Bayesian theories of perceptual decision-making prescribe how previous information should be integrated with current information in a probabilistically optimal manner (Vilares & Kording, 2011). Such theories would predict that current choices should be more strongly biased toward previous choices when the previous choice was associated with high certainty. In line with this idea, several studies have found that choice repetition is stronger when the previous choice was fast and when arousal was low (Braun et al., 2018; Urai et al., 2017)—two factors that have been linked to increased decision confidence (Sanders, Hangya, & Kepecs, 2016; Urai et al., 2017). Moreover, recent studies using a continuous estimation tasks found that a higher self-reported decision confidence on the previous trial was associated with a stronger bias on the current trial toward the previous perceptual estimate (Samaha, Switzky, & Postle, 2019; Suárez-Pinilla, Seth, & Roseboom, 2018). Thus, broadly in line with Bayesian theories, it appears that high decision confidence on the previous trial leads to a stronger choice repetition bias.

Surprisingly, however, it has also been reported that observers are more likely to repeat a previous choice that was based on low, compared to high, sensory evidence (Akaishi et al., 2014). This pattern also occurred when an irrelevant intervening stimulus was shown between choices, ruling out low-level sensory adaptation as an explanation. According to Akaishi and colleagues, choice repetition arises from internal signals as previous choices shift the internal choice estimate, biasing the subsequent choice. They argued that the estimate is updated more toward a choice based on low sensory evidence.

Crucially, there is an apparent contradiction: Akaishi et al. (2014) found that choice repetition was largest when the previous choice was associated with *low* confidence (inferred from low sensory evidence), whereas Urai et al. (2017) and Braun et al. (2018) found that choice repetition was largest when the previous choice was associated with *high* confidence (inferred from fast responses and low arousal). In other words, although strong sensory evidence is associated with high confidence on average, choice repetition is smallest after strong sensory evidence and largest after high confidence. It is unknown whether these paradoxical findings can be attributed to variation in confidence within levels of evidence or whether it must be attributed to other causes. For example, other elements of the previous trial, such as the direction of evidence (i.e., whether the previous physical stimulus evidence was in favor of one or the other interpretation), may play a role in addition to the role of the previous choice. That is, there may be several, possibly interacting, factors that jointly determine the presence and strength of serial choice biases.

We set out to isolate the effects of choice history and evidence history on serial choice bias by examining how different factors modulate choice repetition probability. Participants performed a motion coherence discrimination task, identifying test stimuli as either more or less coherent than a reference stimulus, while also reporting their subjective decision confidence. Stimulus evidence was parametrically varied using six levels of evidence strength. This allowed us to examine the effect of previous decision speed and previous decision confidence, as well as previous stimulus evidence, on choice repetition.

We found that choices were biased toward the previous choice, and that this bias was stronger for confident, as well as fast, previous choices. This is in line with previous findings using response times and pupil dilation as proxy measures for confidence (Braun et al., 2018; Urai et al., 2017). In addition, we found that choices were biased away from the direction of evidence on the previous trial, more so when the evidence was strong, in line with the findings of Akaishi et al. (2014). Taken together, perceptual choices are biased toward the previous choice, a modulation that grows with previous decision confidence, and biased away from the previous evidence direction, a modulation that grows with previous evidence strength. These findings suggest that previous choices and previous stimuli may induce biases on separate stages of perceptual decision-making.

## Methods

### Data availability

All data and code used for stimulus presentation and analysis are available from the Donders Institute for Brain, Cognition and Behavior repository at https://doi.org/10.34973/1wad-3171.

### Participants

Thirty-eight naïve participants (23 female/15 male; age range, 18–34 years) recruited through the university pool took part in the experiment. Subjects were paid 8 euros an hour for their participation. All participants reported normal or corrected-to-normal vision and provided written informed consent before the start of the study. The study was approved by the local ethical committee (CMO region Arnhem-Nijmegen, The Netherlands) and was in accordance with the tenets of the Declaration of Helsinki.

We performed an a priori power analysis that resulted in requiring *n* = 34 to obtain 80% power for detecting at least a medium effect size (*d* ≥ 0.5) with a two-sided paired *t*-test at an alpha level of 0.05. Four participants were excluded from our original sample: one did not complete all sessions, one was excluded after training due to failure to follow task instructions, and two were excluded due to technical errors during the experiment. These participants were replaced with new participants.

### Apparatus and stimuli

Visual stimuli were generated with the Psychophysics Toolbox (Brainard, 1997; Kleiner, Brainard, Pelli, Ingling, Murray, & Broussard, 2007; Pelli, 1997) for MATLAB 2018a (MathWorks, Natick, MA). They were displayed on a 24-in. flat-panel display (BenQ XL2420T, 1920 ×  1080 resolution, 60-Hz refresh rate; BenQ, Taipei, Taiwan). Participants viewed the stimuli from a distance of approximately 70 cm in a dimly lit room.

All stimuli were random dot kinematograms composed of 769 white dots on a black screen, moving within a central circular aperture (12° visual angle radius). The dot density was 1.7 dots per degree^2^. A red fixation cross was displayed at the center of the screen at all times. The population of dots was split into signal dots and noise dots. The signal dots moved in the motion direction of the trial with a velocity of 11.5°/s. If signal dots left the aperture, they were redrawn on the opposite side. Three different sequences of dot motion (at the same coherence and direction) were presented in an interleaved fashion, making the effective speed of signal dots 3.83°/s. The noise dots changed position randomly from frame to frame. The percentage of signal dots defined the motion coherence, a measure of motion strength.

### Procedure

**Figure 1.**
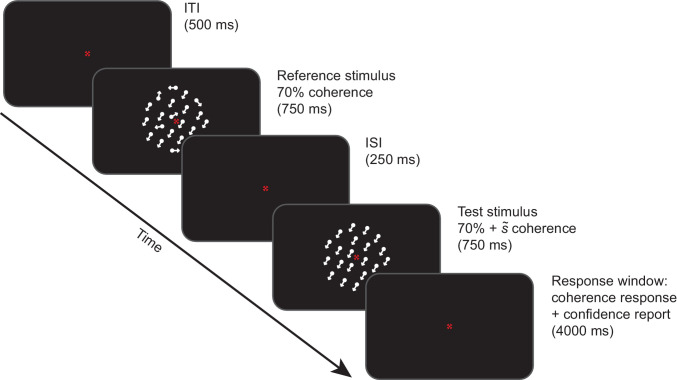
Trial design of main task. A reference random dot motion stimulus of 70% coherence was presented at fixation, followed by a test stimulus with a different coherence but with the same mean motion direction. Participants gave two responses. They first indicated whether the test stimulus had higher or lower coherence than the reference, using the “J” and “K” buttons on the keyboard. They then reported their confidence on a scale of 1 to 4. If they failed to give both responses, they received auditory feedback during the inter-trial interval.

In each trial of the experiment (Figure 1), two white random dot motion stimuli were presented on a black background successively for 750 ms, separated by a 250-ms interstimulus interval. The first stimulus was always a reference stimulus of 70% motion coherence. The second stimulus was a test stimulus with a higher or lower motion coherence than the reference. The difference in motion coherence between reference and test stimuli was taken from one of three sets, chosen on a participant-by-participant basis (procedure described below): easy (1.25%, 2.5%, 5%, 10%, 20%, and 30%), medium (0.625%, 1.25%, 2.5%, 5%, 10%, and 30%), or hard (0.3125%, 0.625%, 1.25%, 2.5%, 5%, and 20%). Both stimuli had the same mean motion direction, and the motion direction of any given trial was randomly offset between 30° and 330° from the motion direction of the previous trial.

Participants were asked to give two responses in order. First, they indicated whether the test stimulus had lower or higher coherence than the reference (coherence response), and, second, they reported how confident they were about their decision on a scale from 1 to 4 (confidence report). For the coherence response, they used their right hand to press either the “J” or “K” button on the keyboard. The button mapping for indicating lower or higher coherence of the test stimulus was counterbalanced across participants. For the confidence report, participants used their left hand to press the corresponding 1 to 4 digit buttons on the left-hand side of the keyboard. Participants had 4.75 seconds to give both responses, starting from the onset of the test stimulus. If they failed to give both responses in the correct order within the time limit, they received auditory feedback consisting of a low tone, played through headphones during the inter-trial interval. The sequence of coherence differences between the reference and test stimuli was pseudorandomized across trials, such that every coherence difference was preceded equally often by every other coherence difference (Brooks, 2012).

Participants completed three sessions—one practice session and two data collection sessions. During the practice session, participants received instructions about the coherence discrimination task and performed one or more simplified practice blocks of 48 trials each, in which they only had to judge the coherence difference without rating their confidence. Next, a staircasing procedure was used to estimate an individual threshold of 70% accuracy in the coherence discrimination task using the QUEST algorithm (Watson & Pelli, 1983). Participants completed at least three blocks of 48 trials each, after each of which the convergence of the threshold estimate was visually inspected. Based on the resulting threshold, one of the three stimulus sets was chosen; for thresholds below 5% and below 10% coherence difference, the hard and medium stimulus sets were selected, respectively. As a result, two participants were assigned the easy set, 22 participants the medium set, and 10 participants the hard set. After the staircasing procedure, participants received instructions for the additional confidence report and practiced the complete task with their stimulus set for the rest of the first session (nine blocks of 48 trials, 432 trials total). The two data collection sessions started with one refresher block of 48 trials. Participants then completed 15 main blocks of 48 trials for each session, resulting in 1440 total trials per participant.

Participants received auditory feedback about the correctness of their decision during the practice blocks and refresher blocks only. This feedback consisted of a brief high or low tone for correct and incorrect decisions, respectively, played through headphones during the inter-trial interval. Participants always received on-screen written feedback about their general performance (percentage correct, average response time, and missed trials) in each block.

### Data cleaning

Trials in which one or both responses were missing and trials where participants gave coherence responses within ≤300 ms from the onset of the test stimulus were removed from further analyses. Consequently, 184 out of 48,960 trials (0.38% of all trials) were discarded.

### Deriving choice repetition from psychometric functions

In order to quantify the choice repetition bias and its modulation by previous evidence, response time, and confidence and to qualitatively compare our findings to those of previous studies, we first employed a psychometric function fitting approach. We estimated choice repetition independently for each condition, following the analytical approach of earlier studies (Akaishi et al., 2014; Urai et al., 2017).

**Figure 2.**
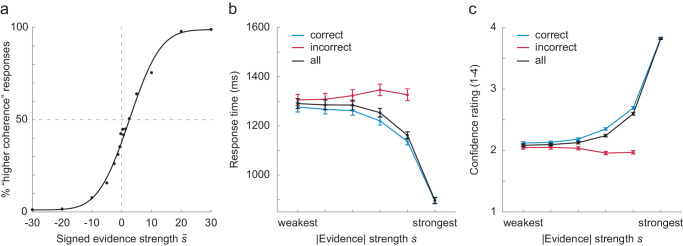
Coherence discrimination performance. (a) Group average responses followed a psychometric function, with a general bias toward lower coherence responses. (b) Mean reaction times decreased with absolute evidence for correct choices and increased for incorrect choices. (c) Mean subjective confidence ratings increased with absolute evidence and decreased for incorrect choices. Error bars represent between-subject *SEM*s. Only data points that contained at least 10 subjects with at least 10 trials are shown.

We first expressed the probability of a higher coherence response, *P*(*r_t_* = 1), as a function of the signed coherence difference between the reference and test stimulus ($\tilde{s_{t}}$) and fit a psychometric function (Figure 2a) (Wichmann & Hill, 2001) of the form

$$P\left(r_{t}=1|\tilde{s_{t}}\right)=\lambda +\left(1-2\lambda \right)g\left(\delta +\alpha \tilde{s_{t}}\right)$$

where *λ* is the probability of stimulus-independent errors (lapses), *g* is the logistic function, *α* is perceptual sensitivity, and *δ* is a bias term. The free parameters *λ*, *α*, and *δ* were estimated by minimizing the negative log-likelihood of the data (using the MATLAB fminsearchbnd function).

For the quantification of serial choice bias, we first split the data into two bins corresponding to the previous choice such that one bin contained all trials for which the participant previously reported higher coherence, and the other bin contained all trials for which they reported lower coherence. For each level of previous absolute evidence strength (*s_t_*_–__1_) within these bins, we further split the data by previous response time (*r_t_*, based on a median split) or previous confidence (low ratings, 1 or 2; high ratings, 3 or 4). For each of those subsets of trials, we fit the psychometric function as described above. In order to compute the choice repetition bias, the resulting bias terms, *δ*, were transformed from log-odds into probabilities by the inverse logit function *P* = *e*^δ^/(1 + *e*^δ^). This probability reflects $P(r_{t}=1|\tilde{s_{t}}=0)$, which is the probability of choosing higher coherence in a hypothetical ambiguous trial (no evidence) in the current trial. To compute choice repetition probabilities, for each bin we averaged the probability to repeat the previous choice across the two previous choice options: $p\left(repeat\right)=\left(p(r_{t}=1|\;r_{t-1}=1,\;\tilde{s_{t}}=0\right)\;+\;p\left(r_{t}=0|\;r_{t-1}=0,\;\tilde{s_{t}}=0\right))\;/\;2$. Finally, to test for differences in choice repetition probability, or *p*(*repeat*), across bins, we performed repeated-measures analyses of variance (ANOVAs) using SPSS Statistics 23 for Windows (IBM, Armonk, NY).

### History-dependent multiple regression model

Although the approach described above allowed us to compare the current results to previous studies on choice repetition, it suffered from the problem that previous trial characteristics, such as evidence strength, response time, and confidence report, are correlated (Figure 2). Splitting data according to one of these variables will partition meaningful variance in the other variables, as well, which can introduce or mask apparent influences of any one variable on choice repetition. Furthermore, because the above analysis was focused on the biasing influence of the previous choice, it is not clear what role the previous stimulus evidence itself plays in biasing subsequent visual processing. To overcome these problems, we devised a history-dependent regression model that allowed us to estimate separate influences of current and previous stimulus evidence variables and response variables.

Specifically, we constructed a generalized linear mixed model (GLMM) with a binomial link function to predict the current choice based on current and previous stimulus evidence and response variables, as well as their interactions. The factors in this regression model can be conceptually split into current-trial factors and history factors, where the current-trial factors describe the stimulus information (i.e., evidence direction, evidence strength, and interactions) on the current trial, and the history factors describe the stimulus information and response characteristics (i.e., choice, response time, confidence report, and interactions) of the previous trial.

We were interested in the influence of the previous choice on current choice. Accordingly, we added the effect of previous choice (*prev choice*) as a factor to the model. To examine whether the influence of the previous choice was larger when participants were confident about that choice, we included an interaction factor (*prev choice* × *prev confidence*) to the model. Similarly, to examine whether the influence of the previous choice was greater when participants had responded quickly, we added another interaction factor (*prev choice* × *prev r_t_*). Furthermore, as the influence of the previous choice could scale with the strength of absolute evidence for that choice (i.e., the coherence difference between the reference stimulus and test stimulus), we included yet another interaction factor (*prev choice* × *prev |evidence|*). Note that these three interactions are all theoretically related to decision confidence: More evidence leads to a more confident decision, just as a lower response time and higher reported confidence reflect a more confident decision.

It is important to note that, due to the difficulty level being staircased, there was an ∼70% correlation of previous choice and previous evidence direction (i.e., the sign of the evidence, determining whether it was a higher coherence or lower coherence trial). This raises the question of whether it is the previous choice or the previous evidence direction that influences the current perceptual decision. To investigate this, we added the previous evidence direction (*prev evidence dir*) to the model, as well as all interactions equivalent to those we included for previous choice. This included interactions with previous confidence (*prev evidence dir* × *prev confidence*), previous response time (*prev evidence dir* × *prev r_t_*), and previous absolute evidence (*prev evidence dir* × *prev |evidence|*, equivalent to the signed evidence of the previous trial).

All factors included thus far describe history effects; however, observers’ decisions are primarily based on the bottom-up information present in the current trial. To account for this, we included the signed evidence of the current trial in the model (*curr evidence dir* × *curr |evidence|*). Finally, we included the main effects of all variables in the aforementioned interactions (with the exception of *prev choice* and *prev evidence dir*, which were already included). Accordingly, we included *prev confidence*, *prev r_t_*, *prev evidence*, and *curr evidence* as factors in the model. Note that these main effects by themselves provide no information about the identity of either the previous or current trial nor information about the previous choice; therefore, they were unlikely to provide information about current choice. Consequently, they were not expected to be significant factors in the model. The reason they were nevertheless added was to prevent an unexpected significant modulation expressing itself as an interaction and hence be misinterpreted.

Before constructing the model, variables were recoded as follows. Categorical predictors *choice* and *evidence dir* were coded using effect coding (–1/1). Confidence was subject-wise centered and subject-wise scaled by its standard deviations. For the response times, we used a robust *z*-score and removed the subject-wise median and scaled by the subject-wise median absolute deviation (constant = 1.48). We scaled the unsigned evidence to range between 0 and 3, to accommodate smaller parameter estimates to prevent numerical floating-number overflow.

We used the R lme4 package (Bates, Mächler, Bolker, & Walker, 2015) to fit a generalized linear model from the binomial family. We fitted a model with “subjects” as the only random grouping factor. We included for each fixed effect its corresponding random slope coefficient, but without random correlations, as the model did not converge. Even with this simplification, the random effect structure was singular, but the model converged according to the lme4 convergence checks. As a robustness check, we re-fit the data with a Bayesian GLMM using brms (Bürkner, 2017; Bürkner, 2018) with an LKJ prior of 2 on the correlation matrix which confirmed all of our findings. For significance testing, we utilized the Wald *Z* test, which is valid only in the asymptotic regime assuming a multivariate normal sampling distribution of parameters and a proportional sampling distribution of the log likelihood to χ^2^. Therefore, we were very conservative in our interpretation of the reported *p*-values when the effects were not obvious from effect sizes alone. An overview of the model output can be found in Supplementary Table S1.

To check whether the model could adequately capture our data, we plotted and compared fitted marginal against aggregated raw marginal data. Mimicking posterior predictive tests, we simulated new datasets from our model and compared the observed simulated data distributions with the observed one. We simulated new responses with the following procedure: For each trial, we took the probability for a more coherent response for this trial as predicted from the GLMM and simulated a coin toss with that probability (Bernoulli trial) to generate a discrete response (0 or 1, test stimulus less or more coherent). We repeated this procedure to create 1000 simulated datasets and compared the patterns in these datasets to the patterns in the empirical data. We generally found our data to be well captured (see Supplementary Figure S1).

## Results

The goal of the current study was to investigate the modulation of sequential choice biases by subjective decision confidence, motivated by the seemingly conflicting roles of previous response times and stimulus evidence. To this end, 34 human observers performed a binary forced-choice coherence discrimination task on random dot motion stimuli. Mean performance across all trials was 70.3% (*SD* = 3.8%). Lapses were infrequent for all participants, as indicated by very high accuracy on trials with the largest coherence difference (98.8%; *SD* = 1.1%). Except for two participants with accuracies of 95.3% and 96.3%, respectively, all participants had over 97% correct responses on these trials. As expected, stronger absolute evidence resulted in higher accuracy (Figure 2a), faster response times (Figure 2b), and higher subjective confidence reports (Figure 2c). In addition, higher confidence reports were associated with higher accuracy and faster response times, which are often considered an implicit measure of decision confidence. These findings suggest that the subjective confidence reports are a meaningful reflection of decision confidence. Both response times and confidence reports exhibited patterns corresponding to decision uncertainty (Sanders et al., 2016). Response times decreased with evidence for correct responses and increased with evidence strength for incorrect responses (Figure 2b), whereas confidence reports increased with evidence strength for correct responses and decreased with evidence for incorrect responses (Figure 2c).

### Choices are biased toward previous choices

Mean choice repetition probability across observers was not significantly different from chance: *p*(*repeat*) *M* = 0.506, *SD* = 0.061; one-sample *t*-test *t*(33) = 0.58, *p* = 0.56. One explanation for this could be idiosyncrasies in sequential choice biases. Many studies have found that, although some observers tend to repeat choices, others tend to alternate (Abrahamyan et al., 2016; Braun et al., 2018; Fründ et al., 2014; Urai et al., 2017; Urai et al., 2019). Because of this variability across observers, choices may not appear systematically biased at the group level. A second explanation is that the choice repetition value *p*(*repeat*) is influenced by covariates, such as the previous evidence direction. To investigate the effect of previous choice on current choice while accounting for other variables, we used a history-dependent regression model (GLMM). The GLMM clearly showed that current choices were biased toward the previous choice (*prev choice*: *b* = 0.25; bootstrapped 95% confidence interval [CI], 0.16–0.35; *p* < 0.001). Moreover, the single-subject parameter estimates of the GLMM revealed little variability in the effect of the previous choice. For 31 out of 34 participants, the *prev choice* parameter was estimated to be positive, signifying an attractive bias; that is, observers repeated their previous choice (Supplementary Figure S2).

### Choice repetition increases after fast choices

**Figure 3.**
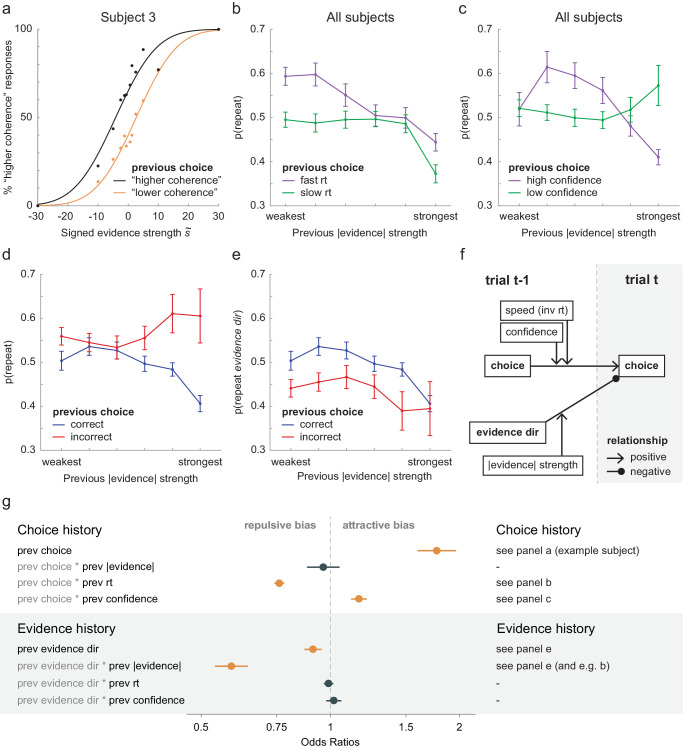
Patterns of choice repetition and GLMM results. All error bars represent between-subject *SEM*s. (a) Responses of an example participant, split for previous choice, revealed a clear choice history bias in this participant. The difference (*δ*) between the curves at $\tilde{s_{t}}=0$ can be converted into a *p*(*repeat*) value using the inverse logit function. (b) Group *p*(*repeat*) values for previous fast response times versus previous slow response times (median split per evidence bin) indicated that choice repetition was higher after fast responses. Paradoxically, choice repetition decreased with previous absolute evidence strength. (c) Group *p*(*repeat*) values for trials with previous high versus low confidence showed a varying modulation of choice repetition by previous confidence. (d) Group *p*(*repeat*) values for previous correct versus previous incorrect choices showed diverging effects of previous evidence strength. Note that the previous choice combined with the correctness of that choice is equivalent to the previous evidence direction. (e) Group *p*(*repeat evidence dir*) values (the likelihood that a choice matches the previous evidence direction) showed that a choice bias away from the direction of evidence on the previous trial increased with previous absolute evidence strength. (f) A schematic representation of the model results depicts how previous trial variables influenced the choice on the current trial. (g) GLMM fixed effects of history factors. Odds ratios < 1 signify a negative estimate, meaning that the higher the term, the lower the current choice (and thus the more likely participants answered “less coherent”); odds ratios > 1 imply a positive estimate. Significant terms (*p* < 0.05) are marked in orange. See Supplementary Table S1 for the full model output.

In line with previous research (Urai et al., 2017), we observed an increase of choice repetition after fast choices (Figure 3b). We confirmed these observed patterns by performing a repeated-measures ANOVA, testing the effect of previous absolute evidence strength and previous response time on the choice repetition values we derived. This revealed a main effect of previous response time, *F*(1, 33) = 32.709 and *p* < 0.001, with increased choice repetition for previous fast compared to slow choices. This main effect of response time was confirmed by our history-dependent regression model, indicating that previous response times negatively modulated the impact of the previous choice on the current choice (*prev choice* × *prev r_t_*, *b* = –0.12; bootstrapped 95% CI, –0.14 to –0.09; *p* < 0.001) (Figure 3g). In other words, participants were more likely to repeat their choice after a fast response.

In addition, our ANOVA revealed a significant interaction between previous response time and previous evidence, *F*(5, 165) = 2.961, *p* = 0.014, indicating that the effect of previous response time was not equal across all levels of previous evidence (see Figure 3b). As we did not have a theoretical motivation to include a *prev choice* × *prev r_t_* × *prev |evidence|* interaction or other three-way interaction in our GLMM, this factor was not included in our original model. However, when we simulated new response data based on the GLMM, we observed a similar fluctuation of the effect of previous response time across different levels of previous evidence (Supplementary Figure S1). This suggests that the interaction in the marginal data, as evaluated with the ANOVA, was driven by other covariates correlated to previous response time and/or previous evidence. Indeed, a separate GLMM with the additional *prev choice* × *prev r_t_* × *prev |evidence|* interaction indicated that the three-way interaction was not significant (*b* = –0.014. *p* = 0.302).

### Choice repetition increases after confident choices

The modulation of choice repetition by response time has been previously interpreted as evidence that choice repetition increases after confident responses. We next sought to test the role of confidence more directly by relating choice repetition to explicit subjective confidence ratings. To this end, we performed a repeated-measures ANOVA on the pattern observed in Figure 3c, testing the effect of previous confidence rating and previous absolute evidence strength on the choice repetition values we derived. This showed a complexly varying modulation of choice repetition by previous subjective confidence across different levels of previous evidence strength: there was an interaction of previous confidence and previous evidence strength *F*(5,165) = 7.918, *p* < .001, but no main effect of previous confidence *F*(1,33) = .444, *p* = .510 (Figure 3c). It is likely that the effect of previous confidence was difficult to derive from this analysis because of multicollinearities in the data. The history-dependent regression model accounts for this and clearly revealed that previous confidence positively modulated the impact of the previous choice on the current choice (*prev choice* × *prev confidence b* = 0.066; bootstrapped 95% CI, 0.03–0.10; *p* < 0.001) (Figure 3g). In other words, participants were more likely to repeat previous choices made with high confidence, even after adjusting for previous response time and previous evidence strength. We found that choice repetition increased following high subjective confidence reports.

### Choice alternation after previous strong stimulus evidence

Next, we investigated whether choice repetition was modulated by previous evidence strength. In line with Akaishi et al. (2014), the psychometric analysis showed that choice repetition decreased after stronger previous evidence strength, with main effect of previous evidence *F*(5, 165) = 13.550, *p* < 0.001 (Figure 3b). This may appear paradoxical in light of our earlier described findings, as trials with strong evidence have faster response times and higher confidence ratings (Figure 2), which would be expected to lead to an increase in choice repetition. Strikingly, our model revealed that choice repetition was not significantly modulated by the strength of the previous evidence (*prev choice* × *prev |evidence| b* = –0.017; bootstrapped 95% CI, –0.10 to 0.05; *p* = 0.662) (Figure 3g), contradicting our previous psychometric analysis. These contradictory findings raise the question of if, and how, the strength of the previous evidence modulates the current choice.

The answer to this question may lie not with the choice history but with the evidence history. When examining choice repetition for previous correct and previous incorrect choices, we found opposite modulations with previous absolute evidence strength. As the previous absolute evidence strength increases, people are more likely to alternate previous correct and more likely to repeat previous incorrect choices (Figure 3d). As the combination of the previous choice and the correctness of this choice is equivalent to the previous evidence direction, this suggests that people are more likely to alternate their choice away from the evidence direction on the previous trial. Indeed, when expressing this probability, *p*(*repeat evidence dir*), we observed a relationship between the current choice and the direction and strength of the previous evidence (Figure 3e).

The GLMM results confirm that current choices, in addition to being biased toward the previous choice, are simultaneously biased away from the previous evidence direction (*prev evidence dir b* = –0.041; bootstrapped 95% CI, –0.08 to 0; *p* = 0.042) (Figure 3g). This is particularly noteworthy because evidence direction and choice were correlated; 70.4% (*SD* = 3.8% across subjects) of choices corresponded to the evidence direction. Yet, the coefficients have opposing signs. We also found that the repulsion away from the previous evidence direction increased when this evidence was stronger (*prev evidence dir* × *prev |evidence| b* = –0.23; bootstrapped 95% CI, –0.30 to –0.15; *p* < 0.001) (Figure 3g). This resembles a classical adaptation effect, which is typically stronger following strong adaptor stimuli (Thompson & Burr, 2009); however, note that in our design observers’ choices are based on the coherence difference between two dot motion stimuli, and it is this difference that induces the observed effect. Consequently, our observed effect is not identical to a classical adaptation effect. These findings reveal that the apparent and puzzling increase of choice repetition with decreasing evidence strength found by Akaishi et al. (2014) (Figure 3b) may in fact not be a modulation of the influence of the previous choice but rather a modulation of the influence of previous sensory evidence on current sensory processing. In summary, we found that choices were biased toward the previous choice, a bias that grew when people were faster and more confident on the previous trial. Simultaneously, choices were biased away from the previous evidence direction, a bias that grew for stronger previous evidence (Figure 3g).

## Discussion

Perceptual choices not only are based on current sensory information but are also systematically biased toward the recent history of previous choices. In the current study, we set out to test how the choice repetition bias is modulated by aspects of the choice history, as well as evidence history. Specifically, we investigated the role of decision confidence on the probability to repeat the same choice on successive trials. Confidence deduced from response times and pupil dilation suggests that people are more likely to repeat previous confident choices (Braun et al., 2018; Urai et al., 2017), in line with an optimal integration of previous and current information in a stable environment. In apparent conflict, confidence deduced from sensory evidence suggests that observers are more likely to alternate from previous confident choices (Akaishi et al., 2014), an effect that cannot be attributed to low-level sensory adaptation. To resolve this conflict, we measured decision confidence with explicit confidence ratings where previous studies probed decision confidence indirectly via response times or pupil dilation.

We found that observers were more likely to repeat confident and fast choices. This is in line with the previous findings from indirect measures of decision confidence and confirms the role of decision confidence in positively modulating choice repetition. Furthermore, we found that choice repetition decreased with increasing evidence strength on the previous trial, in line with Akaishi et al. (2014). Crucially, however, our history-dependent regression model revealed that previous evidence did not modulate the transfer of successive choices but rather the influence of the stimulus evidence of the previous trial, as current choices were found to be biased away from the evidence direction on this previous trial.

### The role of decision confidence in choice repetition

According to Bayesian theories of perceptual decision-making, prior information is integrated with sensory input in a probabilistically optimal manner (Ernst & Banks, 2002; Vilares & Kording, 2011). Such theories would predict that prior information is leveraged more strongly if the uncertainty associated with this information is low; consequently, perceptual choices should be more strongly biased toward previous confident choices. Our findings are in line with these predictions, as we found that people were more likely to repeat previous confident choices. However, we cannot conclude from our data that this integration occurs as described by, and is optimal according to, Bayesian theories.

An open question is what underlying mechanism is modulated by decision confidence. One candidate mechanism would be the process of evidence accumulation, as recent findings show that choice history biases are explained by a history-dependent change in the evidence accumulation (Urai et al., 2019). Confidence could modulate the influence that the previous choice exerts on the slope of evidence accumulation during the formation of subsequent choices. Specifically, confidence could make the slope steeper, thus increasing the likelihood of choice repetition.

Our findings add to the literature describing choice history effects of series of forced choices between two alternatives; however, most perceptual decisions are not binary but rather continuous. In continuous estimation tasks, choices are serially dependent, as observers are biased toward previous choices (Fischer & Whitney, 2014; Fritsche et al., 2017; Samaha et al., 2019; Suárez-Pinilla et al., 2018). It has been shown that these serial dependence biases increase following subjective reports of high confidence (Samaha et al., 2019; Suárez-Pinilla et al., 2018). In line with these findings, when decoding uncertainty (the inverse of confidence) from early sensory areas, behavioral serial dependence is stronger when the previous trial was associated with low uncertainty and the current trial with high uncertainty compared to the reverse situation (van Bergen & Jehee, 2019). Our findings suggest a similar influence of confidence in forced-choice paradigms; however, it is unclear to what extent choice repetition in forced choices and serial dependence in continuous estimations rely on the same underlying processes. More research is necessary to synthesize findings from choice repetition and serial dependence in estimation tasks.

Previous studies of the influence of confidence on choice repetition in forced-choice paradigms have often used proxy measures for confidence, such as pupil dilation, response times, and evidence strength. Notably, a recent study by Lak et al. (2020) reported increased choice repetition biases following decisions based on weak sensory evidence and used this sensory evidence strength as a proxy for decision confidence. In disagreement with the explanation by Lak et al. (2020), that low decision confidence boosts choice repetition, we found that low decision confidence, as measured with subjective reports and reaction times, was clearly associated with a decreased probability to repeat the previous choice. Our current findings point toward an alternative explanation in which sensory evidence strength interacts with stimulus rather than choice history and highlight the potential pitfalls when inferring decision confidence from stimulus properties.

In this study, we assessed confidence using a subjective report measure and also measured response times, and we found that both modulate choice repetition. An open question that remains is whether subjective confidence and confidence as assessed with response times make independent contributions to choice repetition probability. It should be noted that, in our data, confidence reports and response times were correlated with each other and were both correlated with evidence strength, which modulated the influence of the previous stimulus evidence on the current choice. One may wonder whether this multicollinearity in our data affects the interpretability of our model estimates; however, the reported confidence intervals of the parameter estimates suggest that the influence of these variables is robust, pointing toward the interpretation that all of these variables make independent contributions to choice repetition modulation. Further research must be conducted to investigate this interpretation.

### The role of evidence history in choice repetition

At first glance, our finding that observers’ choices are biased away from the previous evidence direction and that this bias grows with evidence strength resembles a sensory adaptation effect frequently described in the literature (Kohn, 2007; Thompson & Burr, 2009; Webster, 2004; Webster, 2012; Webster, 2015). However, it is important to consider that in our experimental design observers made judgments about the *difference* between a reference stimulus and a test stimulus.

Our history effects cannot be explained by sensory adaptation to motion. The main reason for this is that we changed the motion direction at least 30° from one trial to the next. The varying motion direction should have prevented any patterns of motion adaptation across trials. Sensory adaptation, however, can explain the within-trial effect of a general response bias toward perceiving the test stimulus as less coherent than the reference stimulus (Figure 2a), as within a trial both stimuli always had the same motion direction.

As our findings resemble but cannot be explained by sensory adaptation, this raises questions regarding from which neural population(s) and at what stage of the decision formation process our adaptation-like modulation arises. We hypothesize that the adaptation to the evidence direction arises not from a population of sensory neurons that encode the motion coherence, but rather from a neural population that encodes the accumulated (difference in) motion coherence. Also, we hypothesize that neural adaptation away from this difference should occur at a stage after this difference is computed from the bottom-up sensory evidence but before this evidence is converted into the final choice. After all, an adaptation to the choice itself would predict choice alternation, whereas we found choice repetition. Follow-up research should investigate these hypotheses about the neural and temporal characteristics of the evidence adaptation we observed.

Some previous research has described both attractive choice biases and repulsive stimulus biases in perceptual decision-making. Fritsche et al. (2017) found in separate orientation estimation tasks that observer's perception of stimuli was repulsed by previous stimuli whereas the observer's choices were attracted toward previous choices. More recently, Fornaciai and Park (2019) showed a repulsive adaptation effect in the absence of an attractive serial dependence effect when they removed the influence of late modulatory feedback by visual backward masking. These findings suggest that attractive and repulsive effects may jointly but independently contribute to perceptual experience. Indeed, we observed that choice history and evidence history biased perceptual experience in opposite directions yet in tandem.

### Choice history biases adapt to the statistical structure of the environment

Although we observed strong and consistent choice repetition biases, other studies have observed alternation biases; for example, a primate study by Lueckmann, Macke, and Nienborg (2018) found that choices were biased away from the previous choice. This was likely due to differences in the stimulus statistics. In the task utilized by Lueckmann et al. (2018), stimuli were likely to alternate, whereas in our task stimuli were uncorrelated. Indeed, Braun et al. (2018) found that observers adjusted both the strength and the sign of their choice history biases to the stimulus environment. When stimulus sequences were dominated by either repetitions or alternations, this was directly reflected by the sign of the choice history bias.

In addition, although we observed a clear choice bias away from the direction of evidence on the previous trial, some studies have reported an attractive bias toward previous stimulus evidence. For example, the aforementioned study by Lueckmann et al. (2018) found that choices were biased toward the previous target. It is possible that choice feedback, which enables observers to derive the true identity of the previous target, plays a role in this. If monkeys employ a win–stay/lose–switch strategy, this strategy, combined with choice feedback, would result in a choice bias toward the previous target.

Furthermore, whereas the literature describes idiosyncrasy in serial choice biases, we found little or no idiosyncrasy in the choice history bias. Several studies (Abrahamyan et al., 2016; Braun et al., 2018; Fründ et al., 2014; Urai et al., 2017, Urai et al., 2019) report observers utilizing different strategies (e.g. win-stay-lose-switch), resulting in some observers displaying choice repetition and others choice alternating biases. In contrast, we found that the effect of the previous choice was in the same direction for virtually all participants. As variation across different tasks has been described (Urai et al., 2019), this may be due to task characteristics, but it may also be due to differences in analyses. Although we made a distinction between the effects of choice history and evidence history, previous literature has often confounded them. When this distinction is ignored in the analysis, the repulsive effect of evidence history can lead to an apparent choice switching bias and mask an underlying attractive dependency on the previous choice. Between-subject variability in the size (rather than sign) of the influence of the previous choice, the previous evidence direction, and the interactions can lead to apparent idiosyncrasies in sequential choice dependencies. Indeed, when we ignored the distinction between the effect of evidence history and choice history in our data, we also observed idiosyncrasy, with approximately half of participants being “repeaters” and half being “alternators.” This is despite the fact that almost all showed an attractive bias toward the previous choice in the GLMM analysis. Future research should strive to make a careful distinction between the effects of choice history and evidence history in their analysis and interpretation.

## Conclusions

We found that perceptual choices were biased toward the previous choice, a modulation that grew with previous decision confidence and previous response times. At the same time, choices were biased away from the direction of previous evidence, a modulation that grew with previous evidence strength. These findings suggest that previous choice and previous stimulus evidence induces separate biases on subsequence choices through distinguishable and complementary mechanisms, pointing toward a complex process of decision formation.

## Supplementary Material

Supplement 1
