# Supplementary material for: Opposite effects of choice history and evidence history resolve a paradox of sequential choice bias
Source: J Vis. 2020 Nov 19;20(12):9. doi: 10.1167/jov.20.12.9 (PMC7683864; doi:10.1167/jov.20.12.9)
Supplement: Supplement 1 [file jovi-20-12-9_s001.pdf]

	Estimate (*b*)	*SE*	95% *CI*s	*z*	*p*	Random effect *SD*
(Intercept)	-0.58	0.13	-0.77, -0.31	-4.3	1.5e-05	0.76
curr |evidence|	0.11	0.071	-0.02, 0.24	1.5	0.12	0.32
curr evidence dir	0.038	0.018	0.00, 0.07	2.1	0.036	0.043
prev |evidence|	-0.028	0.021	-0.07, 0.01	-1.3	0.2	0.071
prev evidence dir	-0.041	0.02	-0.08, 0.00	-2	0.042	0.033
prev choice	0.25	0.043	0.16, 0.35	5.8	6.7e-09	0.22
prev rt	0.014	0.013	-0.01, 0.04	1.1	0.27	0.034
prev confidence	0.0026	0.019	-0.03, 0.05	0.14	0.89	0.052
curr |evidence| : curr evidence dir	2.3	0.13	2.18, 2.58	17	2e-67	0.74
prev |evidence| : prev evidence dir	-0.23	0.038	-0.30, -0.15	-6	1.6e-09	0.043
prev |evidence| : prev choice	-0.017	0.038	-0.10, 0.05	-0.44	0.66	0.00015
prev evidence dir : prev rt	-0.0043	0.011	-0.03, 0.02	-0.39	0.7	0.00084
prev choice : prev rt	-0.12	0.012	-0.14, -0.09	-10	1.1e-24	6e-05
prev evidence dir : prev confidence	0.008	0.018	-0.03, 0.04	0.45	0.65	0.028
prev choice : prev confidence	0.066	0.018	0.03, 0.10	3.7	0.00023	0.019

Supplementary Table 1: GLMM output. 95% confidence intervals are bootstrapped *CI*s.

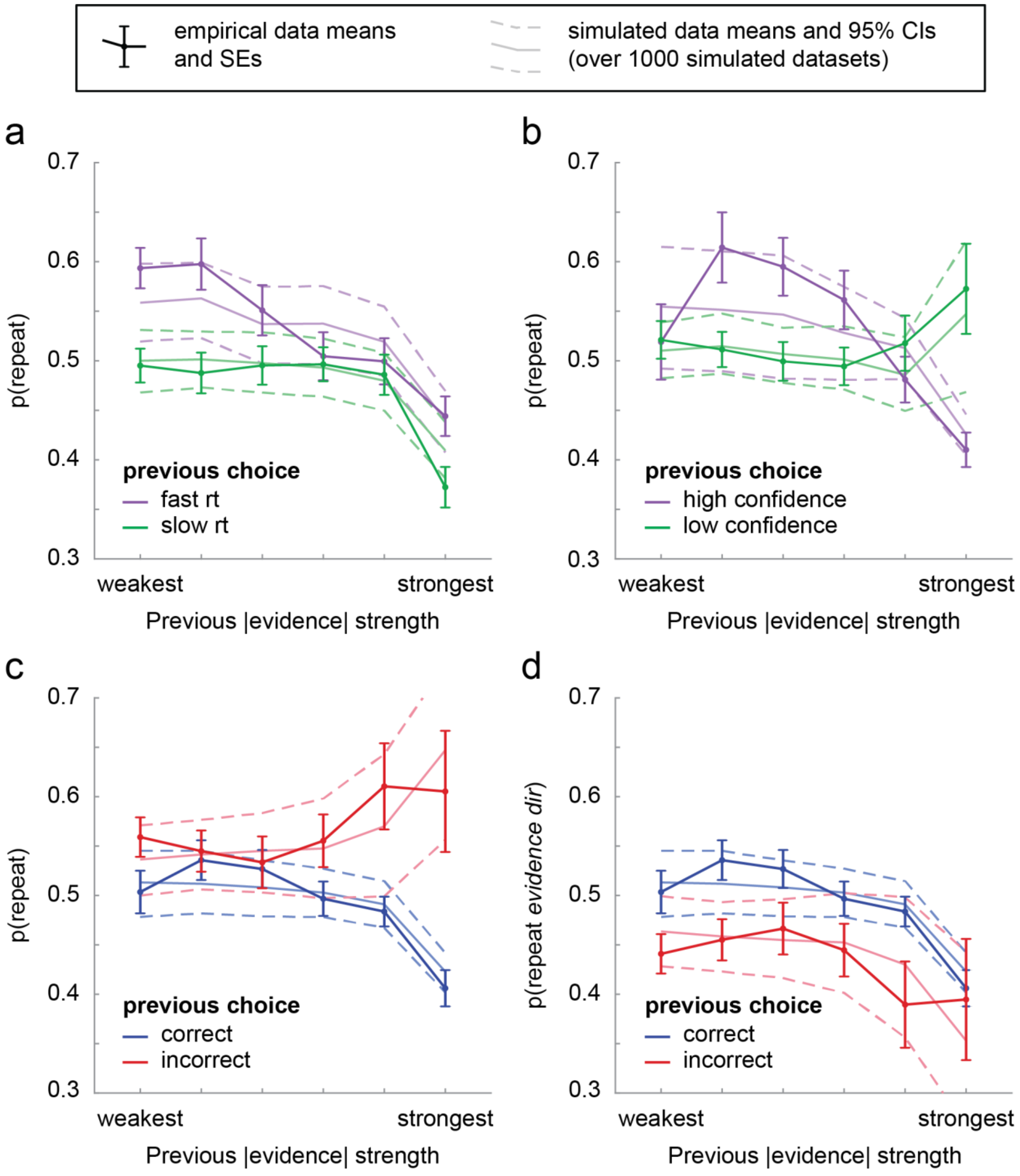

Supplementary Figure 1: a comparison of choice repetition in the empirical data and choice repetition in the 1000 datasets of simulated data based on the GLMM shows that the model is well able to reproduce the patterns in the empirical data. Saturated lines depict empirical data,

with error bars representing SEMs; unsaturated lines depict simulated data, with solid lines representing the means over the datasets and dashed lines representing the 95% CIs. We used a Bernoulli trial procedure to simulate data (see Methods). (a) P(repeat) values for previous fast response times versus previous slow response times (median split per evidence bin) show that choice repetition is higher after fast responses in both real and simulated data. (b) P(repeat) values for previous high and low confidence trials show a varying modulation of choice repetition by previous confidence. (c) P(repeat) values for previous correct versus previous incorrect choices show that choice repetition decreases with previous |evidence| strength after correct choices, but increases after incorrect choices. (d) The probability that the choice matches the previous evidence direction decreases with previous |evidence| strength after both correct and incorrect choices, suggesting that the patterns in (c) may be an effect of the previous evidence, not the previous choice.

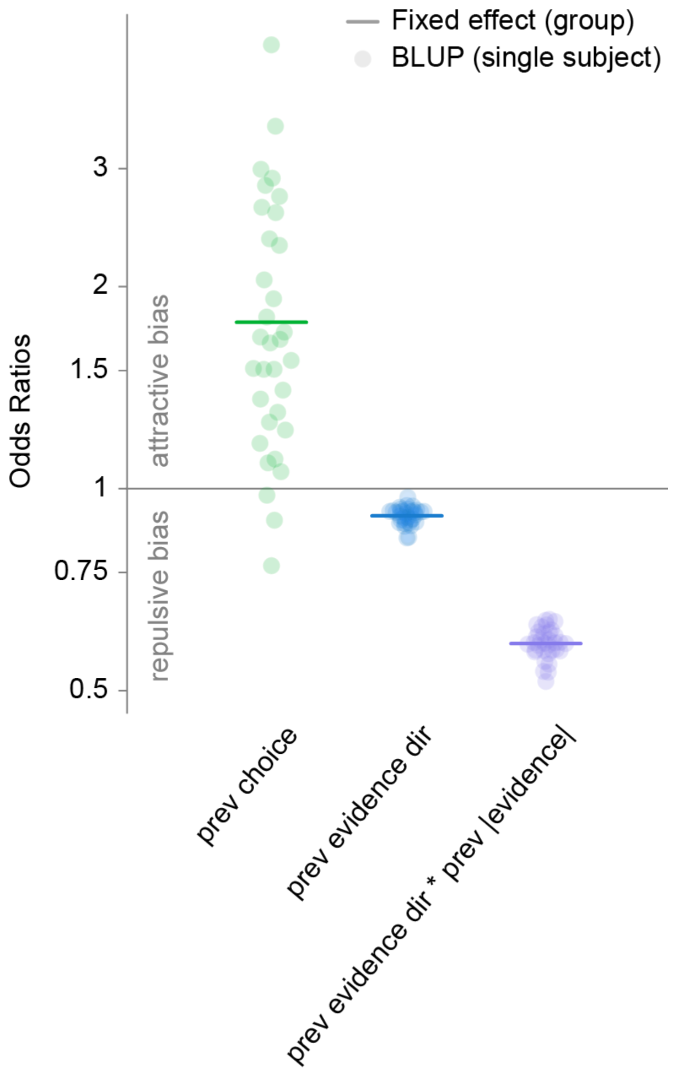

Supplementary Figure 2: BLUP (Best Linear Unbiased Predictions) estimates of the GLMM fixed effects *prev choice*, *prev evidence dir*, and *prev evidence dir * prev |evidence|*. BLUP are single-subject parameter predictions given the model and the data. Consequently, the single-subject parameter predictions are shrunk towards the respective group mean. Odds ratios >1 indicate that the factor has an attractive bias on the current choice, whereas odds ratios <1 indicate a repulsive bias.
